# IMP3 Immunohistochemical Expression in Inverted Papilloma and Inverted Papilloma-Associated Sinonasal Squamous Cell Carcinoma

**DOI:** 10.1155/2021/6639834

**Published:** 2021-02-16

**Authors:** Sarah A. Hakim, Nermine M. Abd Raboh, Lobna S. Shash

**Affiliations:** Department of Pathology, Faculty of Medicine, Ain Shams University, Egypt

## Abstract

Sinonasal inverted papilloma (IP) has a propensity for malignant transformation. Although the IP-associated squamous cell carcinoma (SCC) is rare, it has a poor prognosis. To the best of our knowledge, this is the first study to assess IMP3 immunohistochemical (IHC) expression in sinonasal tumors and to compare it to the Ki-67 IHC expression and to other established clinicopathological parameters. A retrospective study was conducted on three groups which consisted of 72 cases of sinonasal IP, 20 age-matched samples of normal respiratory epithelium, and 15 cases of sinonasal SCC associated with IP, which were obtained from the archives of the Pathology Lab of Ain Shams University Specialized and Ain Shams University Hospitals during the period from January 2012 to December 2019. An IHC study was performed to evaluate IMP3 and Ki-67 expression in the three groups, with correlation of IMP3 expression to established clinicopathological parameters of sinonasal SCC on top of IP. Both IMP3 and Ki-67 showed a sharp rise in expression in the sinonasal SCC group. In addition, there were statistically significant differences in expression values between the 3 groups (*P* = 0.001). Receiver Operating Characteristic (ROC) analysis revealed that IMP3 and Ki-67 could be used to discriminate sinonasal SCC from control and IP lesions, with sensitivity and specificity of 100% and 81.5% for IMP3, respectively, and 100% and 62.5% for Ki-67, respectively. Spearman's rho revealed that both IMP3 and Ki-67 were significantly related to the lymph node and tumor stages but not to the tumor grade. ROC analysis was performed to select cut-off scores for progression and survival for IMP3, and accordingly, Kaplan-Meier analysis showed correlation between IMP3 and overall survival, local recurrence-free survival, and metastasis-free survival in sinonasal SCC cases at the selected cut-off values. Based on our results, IMP3 could serve as a promising diagnostic, prognostic, and therapeutic marker for IP-associated sinonasal SCC.

## 1. Introduction

Sinonasal inverted papilloma (IP) is one of the most common nonmalignant lesions in the nasal cavity and paranasal sinuses. Although benign, it has a high recurrence rate (up to 78% of cases), destroys surrounding structures (including bone erosion and orbital wall involvement), and may undergo malignant transformation (in 5–15% of cases). In fact, sinonasal IPs are concomitantly diagnosed in 1.7–56% of patients with sinonasal squamous cell carcinoma (SCC). Moreover, malignant transformation in such lesions has worse long-term clinical outcomes that include metastasis, postoperative disfigurement, and/or death [1–3]. Thus, precise and early diagnosis, thorough follow-ups, and discovering new prognostic and therapeutic indicators have become necessary in the management of sinonasal IP.

The oncofetal RNA-binding protein insulin-like growth factor 2 mRNA binding protein 3 (IMP3) is expressed during the early stages of embryogenesis and plays a role in cell growth and migration. These functions are mediated by posttranscriptional regulation of cell proliferation via regulation of IGF-2 transcription. After birth, IMP3 becomes epigenetically silenced, such that normal adult tissues show no detectable expression of this protein. However, its reexpression was noted in a series of human malignancies but not in the adjacent normal epithelium [4–6]. Moreover, several studies have demonstrated a prognostic role for IMP3 in several malignancies and their progression [7–13].

Only a few studies have assessed the immunohistochemical (IHC) expression of IMP3 in head and neck SCC [6, 14–18], and none have been conducted on sinonasal tumors. Thus, the primary objectives of the current study were to evaluate the IHC expression of IMP3 in the normal sinonasal epithelium, sinonasal IP, and IP-associated sinonasal SCC and to analyze its expression in relation to Ki-67 being an established biomarker. A secondary objective is comparing the IHC expression of IMP3 to established clinicopathological parameters of SCC on top of IP.

## 2. Materials and Methods

### 2.1. Tissue and Patient Data

The current study was a retrospective study conducted on three groups: 72 cases of sinonasal IP, 20 age-matched samples of normal respiratory epithelium obtained from cases with inflammatory/allergic nasal polypi and cases submitted to exclude fungal infection that proved to be free of fungal infection by periodic acid Schiff, and 15 cases of sinonasal SCC associated with IP. They were obtained from the archives of the Pathology Lab of Ain Shams University Specialized and Ain Shams University Hospitals. These were received and diagnosed during the period from January 2012 to December 2019. All cases underwent surgical intervention such as transnasal endoscopic resection and open surgical resection. The archival files were reviewed to determine the age and sex of patients. Hematoxylin and Eosin (H&E) stained slides were examined to reevaluate and verify the histopathologic diagnosis and histologic grade according to the World Health Organization (WHO) tumor differentiation [19–21]. TNM staging was performed according to the American Joint Committee on Cancer (AJCC) [22]. Normal sinonasal respiratory epithelial samples were also confirmed by histology.

ENT reports of the patients of the third group of IP-associated SCC were reviewed to determine (a) survival time, which was calculated based on the date of major surgery and the date of last follow-up or death, and (b) progression-free survival time, which was calculated based on the date of major surgery or last session of adjuvant postoperative radiotherapy and the date of relapse (local recurrence/distant metastasis) at the last follow-up.

#### 2.1.1. Ethics Statement

Signed written and informed consent was obtained from all participants prior to surgery. The study was approved by the Research Ethical Committee at the Faculty of Medicine, Ain Shams University.

#### 2.1.2. Immunohistochemical Staining

Four-micrometer sections of formalin-fixed and paraffin-embedded samples of normal respiratory epithelium, sinonasal IP, and sinonasal SCC associated with IP were prepared. IHC staining was performed on samples of the three groups using rabbit monoclonal anti-IMP3 (Clone: EP286; Cell Marque, Sigma-Aldrich Co., California, USA; 1 : 100 dilution) and rabbit monoclonal anti-Ki-67 (Clone: SP6; Cell Marque, Sigma-Aldrich Co., California, USA; 1 : 200 dilution). Avidin-Biotin immunoperoxidase complex technique was used by applying the sensitive detection kit (BioGenex, Fermont, California, USA) [23]. This was followed by fixation on poly-L-lysine-coated slides overnight at 37° C. They were then deparaffinized and rehydrated using graded alcohol series. Heating of the sections in a microwave oven in 10 mM citrate buffer (pH 6.0) was performed for 20 minutes. Blocking of endogenous peroxidase, incubation in Protein Block Serum-Free Solution (DakoCytomation, Glostrup, Denmark) for 20 minutes, then incubation overnight at 4°C with primary antibodies were done. Addition of biotinylated anti-mouse immunoglobulin and streptavidin conjugated to horseradish peroxidase was then performed. Finally, 3,3′-diaminobenzidine as the substrate or chromogen was used to form an insoluble brown product. The sections were counterstained with hematoxylin and mounted. Sections of pancreatic ductal adenocarcinoma samples were used as positive control for IMP3 while sections of tonsillar tissue were used as positive control for Ki-67, with each run of every staining procedure performed. Negative control sections of each lesion were incubated with normal mouse serum instead of each of the primary antibodies.

#### 2.1.3. Interpretation of Immunohistochemical Staining

IHC analysis of IMP3 and Ki-67 were blindly performed by three pathologists (the authors). Cytoplasmic staining of IMP3 and nuclear staining of Ki-67 in cells in any of the lesions of the three groups were regarded as positive staining. Slides were scanned by three pathologists at 20x magnification to identify representative hot spots for counting positive cells. The counting of cells with cytoplasmic positivity for IMP3 and cells with nuclear positivity for Ki-67 was performed at 400x magnification. Next, the proportion of positive cells over the total number of counted cells was independently estimated by each of the three pathologists, and the average was calculated. A multiheaded microscope was used to resolve any discrepancies.

The assessment of the intensity of cytoplasmic staining of IMP3 and the nuclear staining of Ki-67 was carried out by subjective consideration of staining intensity, which was scored as follows: 0: negative, 1: weak positivity, 2: moderate positivity, and 3: strong positivity. Any discrepancies were resolved by consensus using a multiheaded microscope [6].

All readings were performed independently and without any prior knowledge of the clinical or histopathological characteristics of the cases.

### 2.2. Statistical Analysis

Data was tested for normality with a Shapiro-Wilk test and expressed as mean ± standard deviation for parametric numerical data or median (interquartile range) for nonparametric numerical data. Categorical variables were expressed as frequencies and percent. Student's *t* test or Mann-Whitney test was used to compare quantitative data between two groups according to data distribution. ANOVA or Kruskal-Wallis tests with post hoc test were used to compare quantitative data between two and more than 2 group tests according to data distribution. Spearman correlation analysis was used to test the strength of correlation between two variables. The ROC curve was used to evaluate the sensitivity and specificity of IMP3 and Ki-67 in predicting SCC. Multivariate logistic regression analysis was performed for determining the predictors of SCC. A significance level of *P* < 0.05 was used in all tests. All statistical procedures were carried out using SPSS version 20 for Windows (SPSS Inc., Chicago, IL, USA).

## 3. Results

### 3.1. Patients

Data describing age and gender of the three studied groups are demonstrated in Table 1.

Among the 15 cases in the sinonasal SCC group, 2 cases (13.3%) were T1, 4 cases were T2 (26.7%), 4 were T3 (26.7%), and 5 were T4 (33.3%). Eight cases were N0/N1 (53.3%), while the nodal stage of the rest of the cases was N2/N3. Three cases (20%) were grade 1, 10 cases (66.7%) were grade 2, and 2 cases (13.3%) were grade 3.

### 3.2. Immunohistochemical Analysis

**Figure 1:**
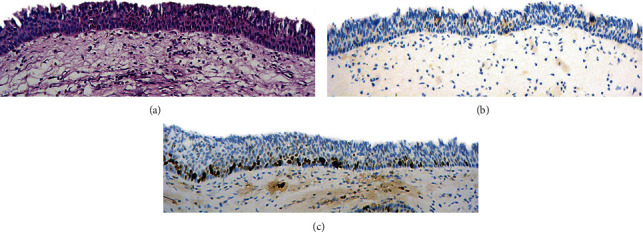
Normal respiratory epithelium: (a) by H&E (H&E ×200); (b) by IMP3 showing low intensity of cytoplasmic staining in scant epithelial cells (IHC ×200); (c) by Ki-67 showing low to moderate nuclear expression in nuclei of epithelial cells (IHC ×200).

**Figure 2:**
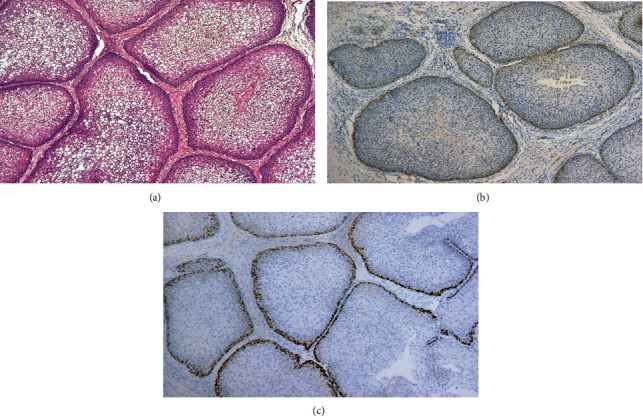
Sinonasal inverted papilloma: (a) by H&E (H&E ×100); (b) by IMP3 showing low intensity of cytoplasmic staining in outermost epithelial cell layer of the IP masses (IHC ×100); (c) by Ki-67 showing scattered nuclear expression of moderate intensity in outermost layer of IP masses (IHC ×100).

**Figure 3:**
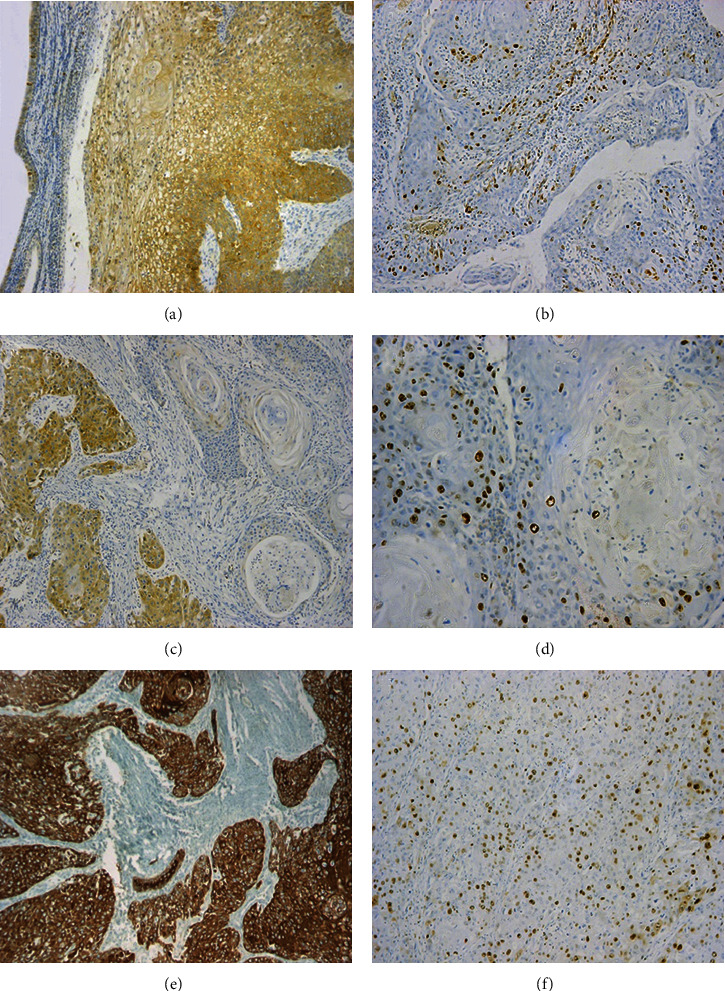
Different cases of IP-associated sinonasal SCC: (a, c, e) IMP3 IHC cytoplasmic expression in tumor cells (IHC ×100); (b, d, f) Ki-67 IHC nuclear expression in tumor cells ((b, f) IHC ×100, (d) IHC ×200).

The overall IMP3 IHC expression revealed a pattern of cytoplasmic expression of low intensity in a few cells of the normal epithelium and sinonasal IP groups. On the other hand, a higher percentage of tumor cells showed IMP3 expression of high intensity among the third group of IP-associated SCC. Ki-67 IHC expression in the normal epithelium and sinonasal IP was characterized by nuclear staining of low to moderate intensity in a few cells. In the sinonasal SCC group, a high percentage of Ki-67 nuclear positivity was detected in the tumor cells (Figures 1, 2, and 3).

**Figure 4:**
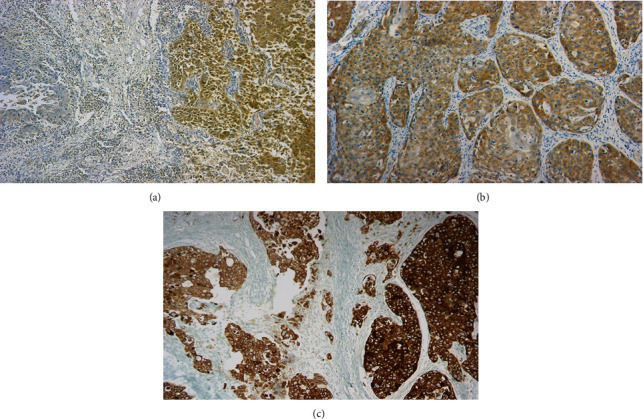
Heterogenous expression of IMP3 in IP-associated sinonasal SCC: (a) in well-differentiated SCC, negative areas of tumor were seen alternating with areas of moderate intensity in well-differentiated SCC (IHC ×100); (b) in moderately differentiated sinonasal SCC, focal strong cytoplasmic staining alternate with foci of cytoplasmic staining of low intensity (IHC ×100); (c) poorly differentiated SCC showed diffuse moderate to strong cytoplasmic staining (IHC ×100).

IMP3 expression was characterized by heterogeneity among SCC cases, such that negative areas alternate with positive areas of low intensity cytoplasmic staining of tumor cells among well-differentiated SCC. This pattern of heterogeneity gradually increases among moderately differentiated cases, until a diffuse cytoplasmic staining of moderate to strong intensity is seen among poorly differentiated SCC (Figure 4).

The mean and median values of IMP3 and Ki-67 IHC expression are presented in Table 2, where both markers show statistically significant differences in expression values between the three groups (*P* = 0.001). Normal epithelium and IP groups had nearly similar values of IMP3 expression while there was a sharp increase in expression among SCC cases. Ki-67 showed a nearly similar pattern of expression as IMP3, with a sharp rise among the sinonasal SCC group (Table 2).

ROC analysis revealed that both IMP3 and Ki-67 could be used to discriminate sinonasal SCC cases from normal/IP cases, with IMP3 at a cut-off value of ≥9.5 with 100% and 81.5% sensitivity and specificity, respectively, and Ki-67 at a cut-off value of ≥22.5 with 100% and 62.5% sensitivity and specificity, respectively (*P* < 0.0001 for each) (Table 3). However, after adjustment of all factors using logistic regression, it was shown that the increase in IMP3 is the only statistically significant factor associated with SCC (OR = 2.4, CI = 1.3–4.5, *P* < 0.01) (Table 4).

### 3.3. Correlation between IMP3, Ki-67, and Clinicopathological Parameters

Both markers showed statistically significant correlation with each other within each of the three groups (*P* = 0.0001 for each) (Table 5).

Spearman's rho analysis revealed that both IMP3 and Ki-67 expressions were significantly related to lymph node and tumor stages, but not significantly related to tumor grade (Table 6).

### 3.4. Survival Analysis

ROC analysis was used to select clinically important cut-off scores of progression and survival for IMP3 in the 15 cases of sinonasal SCC on top of IP. At each percentage score, the sensitivity and specificity for each survival outcome under study were plotted, thus generating a ROC curve. The score having the closest distance to the point with both maximum sensitivity and specificity was selected as the cut-off score leading to the greatest number of tumors which were correctly classified as having or not having the clinical outcome.  Table 7 shows that IMP3 could be used to predict mortality, local recurrence, and distant metastasis in SCC on top of IP at cut‐off levels ≥ 55, ≥31, and ≥49, respectively, with 100% sensitivity and specificity.

**Figure 5:**
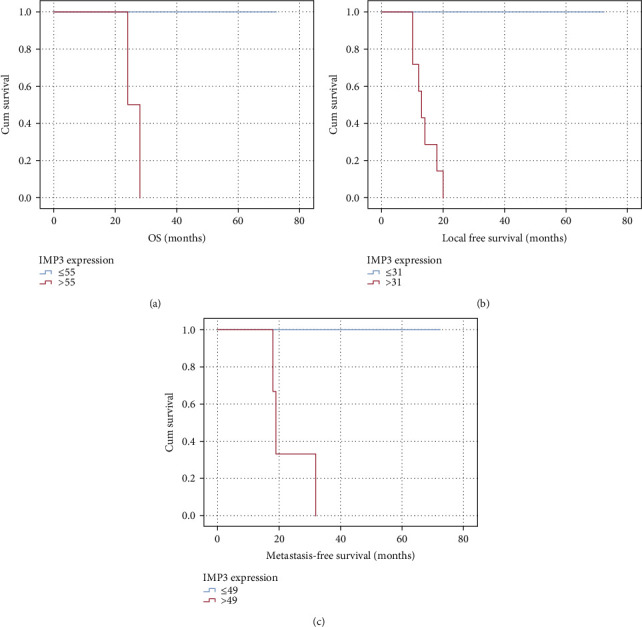
(a) Kaplan-Meier analysis showing correlation between IMP3 IHC expression and OS at a cut‐off level >55; (b) Kaplan-Meier analysis showing correlation between IMP3 IHC expression and local recurrence-free at a cut‐off level >31; (c) Kaplan-Meier analysis showing correlation between IMP3 IHC expression and distant metastasis at a cut‐off level >49.

Kaplan-Meier analysis showed the correlation between IMP3 and overall survival (OS), where OS was 100% at 70 months follow-up at a cut‐off value ≤ 55, whereas it was 0% at 28 months at IMP3 cut‐off >55 (*P* < 0.0001). Local recurrence-free survival (LRFS) was 100% at 70 months follow-up at an IMP3 cut‐off level ≤ 31, whereas it was 0% at 20 months at a cut‐off >31 (*P* < 0.0001). Metastasis-free survival (MFS) was 100% at 70 months follow-up at an IMP3 cut‐off level ≤ 49, whereas it was 0% at 32 months at a cut‐off value >49 (Figure 5).

## 4. Discussion

Although a small number of sinonasal IP are associated with a synchronous or metachronous squamous cell carcinoma, malignant transformation of these lesions leads to long-term morbidity and/or mortality. This necessitates better and novel diagnostic, prognostic, and therapeutic approaches [24]. The oncofetal protein IMP3 has been shown to be expressed in human malignancies but not in nonmalignant lesions, which may serve as a diagnostic marker in such cases. These malignancies include cancers such as ovarian, serous endometrial, cervical adenocarcinomas, pleural, lung, colorectal, gastric, bladder, renal, and laryngeal SCC. On the other hand, other studies have introduced IMP3 as a prognostic biomarker for certain malignant tumors such as those found in the bile duct, neuroendocrine tumors of the lung, and peritoneal mesothelioma [4–12]. However, only a few studies have assessed the role of IMP3 in head and neck tumors [6, 14–18], and none have assessed its expression in sinonasal tumors.

The current study showed that IMP3 and Ki-67 significantly correlated among the three studied groups. Moreover, both proteins had a specific pattern of expression that was nearly similar for the normal epithelium and IP groups and sharply increasing in the IP-associated SCC group. This was in agreement with the pattern described by Maržić et al. [6] for the same markers in laryngeal SCC. However, Humayun and Prasad showed a continuous gradual rise in expression for Ki-67 expression among control, precancerous lesions, and their oral SCC cases [25]. In another study, Altavilla et al. showed a wide range of Ki-67 IHC expressions among 19 cases of sinonasal IP. Increased and diffuse nuclear reactivity was observed among the three cases of IP showing dysplastic changes, one of which was harboring a focal carcinomatous change [26].

With its diverse morphology, IPs may be associated with varying degrees of atypia, dysplasia, carcinoma in situ, and SCC. By computed tomography scan, the sensitivity and specificity of the diagnosis of SCC in IP are low, and orbital wall invasion can be found in recurrent benign IP tumors [27]. Moreover, the histologic criteria of malignant transformation have not yet been well defined. Even the current WHO classification has not included “carcinoma ex sinonasal papilloma” among its topics, which might negatively affect the precise diagnosis of such a category [28]. It should also be noted that the IHC profile of this group is yet to be established, despite several IHC studies conducted in this context [27, 29–40].

Thus, the results of the current study are novel in this area, where ROC analysis revealed that both IMP3 and Ki-67 could be used to discriminate sinonasal SCC cases from IP cases. Furthermore, logistic regression demonstrated that IMP3 is the only statistically significant factor associated with sinonasal SCC after adjustment of other factors. This was in concordance with a previous study [6], whose results revealed that IMP3 was statistically significant in predicting whether the patient had laryngeal SCC or not.

In the current study, IMP3 sensitivity and specificity in differentiating sinonasal SCC from noncancerous cases were 100% and 81.5%, respectively. This was unlike Maržić et al. and Chen et al. [6, 17], whose cases of laryngeal SCC did not show 100% sensitivity for IMP3 expression. Moreover, the present study showed heterogeneity in IMP3 expression among sinonasal SCC, such that negative areas were seen alternating with areas of low intensity in well-differentiated sinonasal SCC, focal strong cytoplasmic staining alternate with cytoplasmic staining of low intensity in moderately differentiated sinonasal SCC, and diffuse moderate to strong cytoplasmic staining in poorly differentiated sinonasal SCC. This was also recorded by Maržić et al. in laryngeal SCC, Ikenberg et al. in prostatic carcinoma, and Sakakibara et al. in esophageal carcinoma [6, 41, 42]. Whether such heterogeneous patterns reflect the recent assumption of the preservation role of IMP3 or not could be argued [43]. In such sense, IMP3's spatial varying expression patterns might indeed highlight the more potent population of tumor cells capable of survival, metastasis, and recurrence. Nonetheless, this heterogeneity warrants caution in interpretation, especially in small biopsies. Caution was also raised in previous studies [41, 42] because IMP3 showed a sensitivity lower than 100%. On the other hand, the current study demonstrated a more consistent 100% IMP3 sensitivity in detecting sinonasal SCC.

Our current work revealed that IMP3 expression was significantly related to lymph node and tumor stages, but not to tumor grade. This was in agreement with Clauditz et al. [16], who found significant correlation between IMP3 expression in head and neck SCCs and both tumor and lymph node stages, but not tumor grade. Li et al. [15] revealed significant correlation between IMP3 expression in their cases of tongue SCC and lymph node stage but not with tumor stage nor tumor grade.

Guided by Zlobec et al. [44], ROC curves were plotted to detect cut-off scores of survival and progression for IMP3, and accordingly, Kaplan-Meier analysis was conducted which showed that IMP3 correlated with shorter OS, LRFS, and MFS at the estimated cut-off values (*P* < 0.0001). This was partly similar to Li et al. [15] whose cases of tongue SCC showed that IMP3 expression had significantly worse OS and lymph node metastasis. Clauditz et al. [16] also showed that IMP3 correlated with OS. However, unlike the current study, their study revealed no significance between IMP3 expression and recurrence-free survival.

A previous study has demonstrated some promising results that support the use of IMP3 as a potential vaccination therapy in patients with head and neck SCC, which showed an immune response that improved the OS of patients [45]. Another study revealed that local recurrence was the main cause of treatment failure in the sinonasal SCC on top of the IP group and suggested that a more aggressive but local treatment in the early stage may be helpful in improving prognosis in such patients [46].

The correlation between IMP3 expression to OS and higher incidence of local recurrence suggests targeting IMP3 might decrease the incidence of local recurrence, and thereby improve the prognosis of such cases [46].

The current study is the first to evaluate IMP3 IHC expression in three separate groups of samples from the sinonasal tract-normal sinonasal epithelium, IP, and SCC on top of IP. Among the points of strength of the current study was comparing the IMP3 IHC expression to an established biomarker of carcinogenesis, namely, Ki-67. It showed a potential diagnostic role for IMP3 in discriminating SCC on top of IP from noncancerous cases with sensitivity and specificity of 100% and 81.5%, respectively. This might suggest that in problematic cases, IMP3 IHC expression could serve as a low-cost ancillary aid. Nevertheless, more studies including dysplastic changes should be performed.

Moreover, the present study has demonstrated that IMP3 expression correlated with poor prognostic indicators and tumor progression in cases of SCC on top of IP. A potential therapeutic value in such cases could be achieved by targeting IMP3 in order to improve prognosis. However, only multi-institutional prognostic studies, to encompass a larger sample size, would be able to confirm the prognostic and therapeutic impact of IMP3 in this context.

Therefore, a limitation to this study is the small number of patients with IP-associated sinonasal SCC (15 cases). This might be attributed to the rarity of this diagnosis. Hence, we found it intriguing to explore all the data we had and to fully present it as a cornerstone for further studies to build upon.

## 5. Conclusion

This study is one of a few IHC studies investigating IP-associated sinonasal SCC. In addition, it proposed novel insights about the attributes of IMP3 in cancer initiation and progression through evaluation of its IHC expression in normal, benign neoplastic, and frankly malignant sinonasal SCC. Such results not only help in offering a better understanding of sinonasal carcinogenesis but also provide a potential low-cost ancillary tool to resolve enigmatic differential diagnoses with high yield diagnostic accuracy in IP-associated sinonasal SCC detection. Furthermore, in the malignant spectrum, IMP3 might also provide a promising prognostic marker and a potential therapeutic target.

## Figures and Tables

**Table 1:** Description of age and gender among the studied groups.

	Group					
	Normal epithelium		Inverted papilloma		SCC	
	Mean	±SD	Mean	±SD	Mean	±SD
Age	47.95	6.03	48.43	5.42	57.80	9.30
Gender						
Male	9	45.0%	49	68.1%	13	86.7%
Female	11	55.0%	23	31.9%	2	13.3%

SD: standard deviation; SCC: squamous cell carcinoma.

**Table 2:** Comparison between the studied groups with regard to IHC expression of IMP3 and Ki-67 values.

	Group									*P*	Sig
	Normal epithelium			Inverted papilloma			SCC				
	Mean	±SD	Median (IQR)	Mean	±SD	Median (IQR)	Mean	±SD	Median (IQR)		
IMP3	6.86	2.63	6.9 (5.1–8)	6.41	3.1	5.2 (4.6–8)	31.93	22.11	17 (12–48)	0.001^∗^	HS^A^
Ki-67	21.76	7.86	21 (16.5–28.9)	20.05	6.43	19.9 (18–25.5)	31.43	8.55	26 (25–38)	0.001^∗^	HS^B^

^∗^Kruskal-Wallis test. ^A^SCC vs. normal (HS: highly significant), SCC vs. inverted (HS), normal vs. inverted (NS: nonsignificant). ^B^SCC vs. normal (HS), SCC vs. inverted (S: significant), normal vs. inverted (NS).

**Table 3:** ROC curve using IMP3 and Ki-67 to differentiate SCC cases from noncancerous cases.

IHC marker	AUC (CI)	Sensitivity	Specificity	+PV	-PV	*P* value (sig)
IMP3 cut‐off level ≥ 9.5	0.971 (0.918 to 0.994)	100.00	81.52	46.9	100.0	<0.0001 (HS)
Ki‐67 cut‐off level ≥ 22.5	0.828 (0.743 to 0.894)	100.00	65.22	31.9	100.0	<0.0001 (HS)

AUC: area under the curve; CI: confidence interval; +PV: positive predictive value; -PV: negative predictive value; sig: significance; HS: highly significant.

**Table 4:** Logistic regression to study independent factors associated with SCC.

	Odds ratio (OR)	*P*	Sig	95% CI for OR	
				Lower	Upper
Age	1.044	.670	NS	0.857	1.270
Gender (female)^∗^	0.051	.585	NS	0.0041	1.10
IMP3 expression	2.443	.005	HS	1.309	4.560
Ki-67 expression	0.759	.113	NS	.539	1.068

^∗^Reference male. CI: confidence interval.

**Table 5:** Correlation between IMP3 and Ki-67 expression among the three studied groups.

Normal epithelium group		Ki-67 expression
IMP3 expression	*r*	0.737^∗^
	*P*	0.0001
	Sig	HS

Inverted papilloma group		Ki-67 expression
IMP3 expression	*r*	0.818^∗∗^
	*P*	0.0001
	Sig	HS

Sinonasal SCC		Ki-67 expression
IMP3 expression	*r*	0.935^∗∗^
	*P*	0.0001
	Sig	HS

^∗^Pearson correlation; ^∗∗^Spearman's rho.

**Table 6:** Correlation between IMP3 and Ki-67 expression and clinicopathological parameters of SCC cases.

		IMP3 expression	Ki-67 expression
Lymph nodal stage	Rho	0.842^∗^	0.788^∗^
	*P*	0.0001	0.0001
	Sig	HS	HS

Tumor histologic grade	Rho	0.488	0.364
	*P*	0.065	0.182
	Sig	NS	NS

Tumor stage	Rho	0.596^∗^	0.572^∗^
	*P*	0.019	0.026
	Sig	S	S

^∗^Spearman's rho.

**Table 7:** ROC analysis using IMP3 to predict mortality, local recurrence, and distant metastasis among SCC cases.

IMP3	AUC (CI)	Sensitivity	Specificity	+PV	-PV	*P* (sig)
Overall survival cut‐off level ≥ 55	1.0 (1.0 to 1.0)	100.00	100.00	100.00	100.00	<0.027 (S)
Local recurrence cut‐off level ≥ 31	1.0 (1.0 to 1.0)	100.00	100.00	100.00	100.00	<0.001 (HS)
Distant metastasis cut‐off level ≥ 49	1.0 (1.0 to 1.0)	100.00	100.00	100.00	100.00	<0.009 (HS)

## Data Availability

All data generated or analyzed during this study are included in this published article.
